# Dent-2 disease with a Bartter-like phenotype caused by the Asp631Glu mutation in the OCRL gene

**DOI:** 10.1186/s12882-022-02812-9

**Published:** 2022-05-12

**Authors:** Eleni Drosataki, Sevasti Maragkou, Kleio Dermitzaki, Ioanna Stavrakaki, Dimitra Lygerou, Helen Latsoudis, Christos Pleros, Ioannis Petrakis, Ioannis Zaganas, Kostas Stylianou

**Affiliations:** 1 Nephrology Department, Heraklion University Hospital, Voutes, 71500 Heraklion, Crete Greece; 2 Foundation for Research and Technology – Hellas, Heraklion, Crete Greece; 3 Department of Nephrology, Saarland University Medical Center, Homburg, Germany; 4 Neurogenetics Laboratory Medical School, University of Crete, Heraklion, Greece

**Keywords:** Dent disease, Bartter syndrome, OCRL, Case report

## Abstract

**Background:**

Dent disease is an X-linked disorder characterized by low molecular weight proteinuria (LMWP), hypercalciuria, nephrolithiasis and chronic kidney disease (CKD). It is caused by mutations in the chloride voltage-gated channel 5 (*CLCN5*) gene (Dent disease-1), or in the *OCRL* gene (Dent disease-2). It is associated with chronic metabolic acidosis; however metabolic alkalosis has rarely been reported.

**Case presentation:**

We present a family with Dent-2 disease and a Bartter-like phenotype. The main clinical problems observed in the proband included a) primary phosphaturia leading to osteomalacia and stunted growth; b) elevated serum calcitriol levels, leading to hypercalcemia, hypercalciuria, nephrolithiasis and nephrocalcinosis; c) severe salt wasting causing hypotension, hyperaldosteronism, hypokalemia and metabolic alkalosis; d) partial nephrogenic diabetes insipidus attributed to hypercalcemia, hypokalemia and nephrocalcinosis; e) albuminuria, LMWP.

Phosphorous repletion resulted in abrupt cessation of hypercalciuria and significant improvement of hypophosphatemia, physical stamina and bone histology. Years later, he presented progressive CKD with nephrotic range proteinuria attributed to focal segmental glomerulosclerosis (FSGS). Targeted genetic analysis for several phosphaturic diseases was unsuccessful. Whole Exome Sequencing (WES) revealed a c.1893C > A variant (Asp631Glu) in the *OCRL* gene which was co-segregated with the disease in male family members.

**Conclusions:**

We present the clinical characteristics of the Asp631Glu mutation in the *OCRL* gene, presenting as Dent-2 disease with Bartter-like features. Phosphorous repletion resulted in significant improvement of all clinical features except for progressive CKD. Angiotensin blockade improved proteinuria and stabilized kidney function for several years.

**Graphical abstract:**

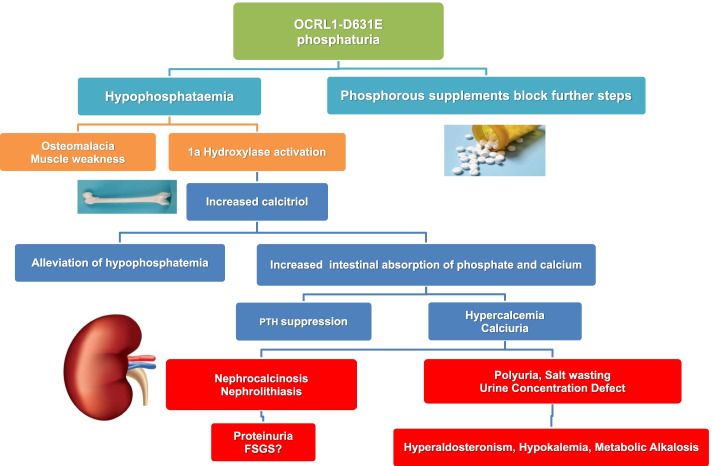

## Background

Dent disease is an X-linked disorder of proximal renal tubules characterized by low molecular weight proteinuria (LMWP) and hypercalciuria with or without nephrocalcinosis. Other signs, that appear with variable frequency and may be helpful for the clinical diagnosis include nephrolithiasis, hematuria, hypophosphatemia, or chronic kidney disease (CKD) [1, 2]. Thirty to 80% of affected males progress to end stage renal disease (ESRD) between ages 30 and 50 years [1, 3]. Rickets, osteomalacia and short stature are occasionally observed. A pathogenic variant in *CLCN5* accounts for 60% of those with Dent disease (known as Dent disease-1, DD1) whereas a pathogenic variant in the *OCRL* gene accounts for another 15% (known as Dent disease-2, DD2) [4]. In the rest 25%, pathogenic variants cannot be confirmed so far [1].

We present a patient with DD2 presenting with unusual features of severe metabolic alkalosis and salt wasting in the urine, mimicking Bartter syndrome. We found an *OCRL* mutation that co-segregated with the disease in male family members. No Bartter associated mutations were found. Interestingly, phosphate repletion resulted in an abrupt and impressive resolution of hypercalciuria and clinical improvement in the long term.

## Case presentation

The index patient was 28-year-old Caucasian man, with a history of stunted growth, bone fractures, hypokalemia, hypercalcemia, nephrocalcinosis and nephrolithiasis. He had first sought medical attention at age 25 when he presented with renal colic and was diagnosed with kidney stones and nephrocalcinosis.

He complained of muscle and bone aches, polyuria and polydipsia. Physical examination revealed postural hypotension, dehydration and several keloids after minor injuries. Medullary sponge disease was excluded by an intravenous pyelogram and a computed tomography. Blood tests showed mild hypernatremia, hypokalemia, metabolic alkalosis, markedly elevated plasma renin and aldosterone, mild hypophosphatemia, hypercalcemia and normal renal function (Table 1). Spot urine samples were consistently alkaline with mild (1+) proteinuria and low specific gravity. Urine biochemistry revealed sodium wasting, accounting for dehydration and postural hypotension, potassium wasting, increased fractional excretion (FE) of uric acid (17%), increased FE of phosphorous (14–24%) and hypercalciuria. The ratio of tubular maximum reabsorption of phosphate over glomerular filtration rate (TmP/GFR) was consistently below normal range. Serum creatine phosphokinase and lactic dehydrogenase were normal. Urine protein was increased (0.5 g/day) and comprised mostly albumin. Urine b2-microglobulin was slightly elevated. PTH levels were at the lower normal limit, 25-OH-Vitamin-D was low, 1–25-(OH)2-Vitamin-D was elevated and urine cyclic adenosine monophosphate (cAMP) was low. A bone biopsy showed reduced volume of cortical and trabecular bone and increased osteoid, compatible with osteomalacia and osteopenia.

**Table 1:** Results of serum and urine laboratory investigations in various measurements in the index patient

Laboratory values	Observed values (range)	Normal values
Hematocrit (%)	46–52	42–49
Serum Sodium (mEq/L)	**145–149**	140–145
Serum Potassium (mEq/L)	**3.2–3.6**	3.5–4.6
Serum cCalcium (mg/dL)	**10.4–11.3**	8.2–10.5
Serum Phosphorous (mg/dL)	**2.1–2.6**	2.5–4.2
Serum Parathormone (pg/mL)	23	10–65
Arterial pH	**7.43–7.55**	7.35–7.45
Arterial HCO3 (mEq/L)	**27–40**	24–25
Serum Creatinine (mg/dl)	0.7	0.6–1.2
Urine Sodium (mEq/day)	224	
Urine Potassium (mEq/day)	109	
Urine Chloride (mEq/day)	200	
Urine Calcium (mg/day)	**347**	< 250
Urine Calcium/body weight (mg/day/kg)	7	< 4
Urine Phosphorous (mg/day)	476	
Urine Uric acid (mg/day)	1472	< 750
Urine protein (mg/day)	**700–1500**	< 250
Urine Oxalate (mg/day)	**92**	7–44
Urine b2-microglobulin (mg/l)	**0.48–0.98**	< 0.2
Urine FE phosphorous (%)	**14–24**	< 15
Urine FE HCO3 (%)	1.7%	
TmP/GFR (mg/dL)	**1.8–2.5**	2.5–4.5
25(ΟΗ) Vit-D (ng/ml)	**8**	10–60
1,25(ΟΗ)2 Vit-D (pg/ml)	**64**	18–62
Urine cAMP (nmol/dl)	0.9	1.6–6.2
Serum renin (mIU/l)	**275**	5–47
Serum aldosterone (pmol/L)	**669**	20–130

A water deprivation test revealed partial nephrogenic diabetes insipidus. Urine acidification was intact and glycosuria was absent. Eye examination was unremarkable.

Summarizing the main clinical features observed in the proband, included: 1) Salt wasting causing hypotension, hyperladosteronism, hypokalemia and metabolic alkalosis. Metabolic alkalosis was accompanied by elevated urine calcium and chloride levels similar to those found in Bartter’s syndrome; 2) phosphaturia and hypophosphatemia leading to elevated calcitriol and osteomalacia; 3) hypercalcemia due to increased calcitriol synthesis leading to hypercalciuria, lithiasis and nephrocalcinosis; 4) partial nephrogenic diabetes insipidus attributed to nephrocalcinosis, hypercalcemia and hypokalemia; 5) albuminuria and LMWP; 6) hypouricemia, hyperuricosuria and hyperoxaluria.

Based on the above considerations the proband was treated with potassium and phosphorous (1 g/day) supplements orally, that were well tolerated. Three years later a second bone biopsy showed marked improvement in bone pathology. The body weight increased from 53kgr to 63kgr, physical stamina had improved, musculoskeletal pains, kidney colics and hypercalciuria had resolved, and electrolyte abnormalities had waned off. A new ultrasound showed stabilization of nephrocalcinosis and absence of kidney stones. More importantly, his everyday life improved after treatment.

In the differential diagnosis we considered phosphaturic syndromes such as hereditary hypophosphatemic rickets with hypercalciuria. Due to the presence of proteinuria, Dent disease was also a consideration, although b2-microglobulin was marginally elevated in the initial work up. However, it was substantially increased 3 years later (5 times the upper normal limit). Targeted mutation analysis did not reveal any mutations in the chloride voltage-gated channel 5(*CLCN5*), the sodium-hydrogen exchanger regulatory factor (*NHERF1*) and the solute carrier family 34 member-1 (*SLC34A1)* and member-3*,* (*SLC34A3).* A syndrome of elevated serum phosphatonins was excluded based on the elevated calcitriol levels.

**Fig. 1:**
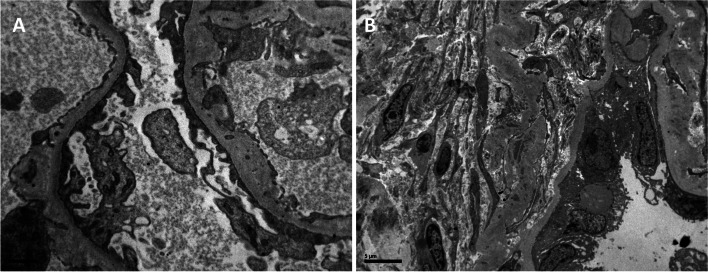
Electron microscopy of kidney biopsy. A) Focal fusion of podocyte foot processes and focal thickening of glomerular basement membranes; B) interstitial fibrosis and thickening of tubular basement membranes. Bars represents 5μm length

Four years after admission he developed nephrotic range proteinuria (4 g/day) and the estimated glomerular filtration rate (eGFR) progressively declined to 65 ml/min/1.73m^2^. At this point, phosphate and potassium supplements were withdrawn. A renal biopsy revealed a FSGS pattern of injury, mild interstitial fibrosis and interstitial calcium deposits with rupture of tubular basement membranes (TBMs). Electron microscopy showed focal fusion of podocyte foot processes and marked thickening of TBMs (Fig. 1). Institution of an angiotensin receptor blocker, improved proteinuria and stabilized renal function for some years. However currently, i.e., fourteen years after initial presentation, proteinuria and eGFR have reached 3 g/day and 30 ml/min/1.73m^2^ respectively.

**Fig. 2:**
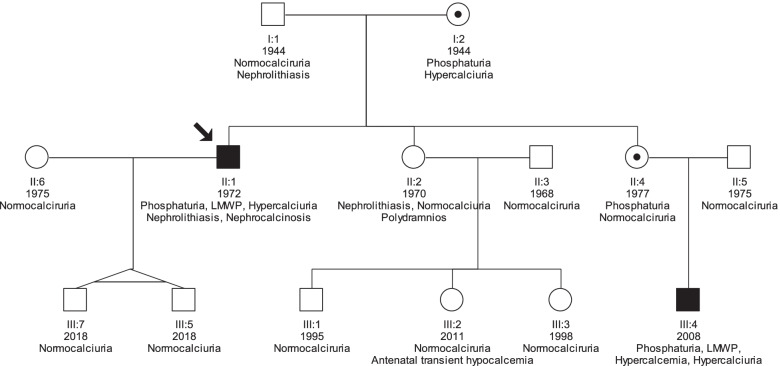
Family tree and segregation analysis for the Asp631Glu mutation of OCRL1. Family members were examined for the Asp631Glu mutation; two males and two females were affected. Males presented the whole spectrum of Dent-2 disease. Female carriers presented only phosphaturia. LMWP: low molecular weight proteinuria

To resolve the conundrum and clarify the underlying pathophysiology, we obtained written informed consent from all family members and proceeded to perform whole exome sequencing (WES), along with clinical and laboratory examination. This analysis identified the c.1893C > A variant in the exon 18 of the *OCRL* gene, (Asp631Glu, rs754567476) as a potential cause for the disease phenotype. Next, we verified the mutation by Sanger sequencing and genotyped all family members (12 out of 14) except the two newborn proband’s twin sons. The Asp631Glu mutation, was segregated with the disease in this family (Fig. 2); the two hemizygous males, presented phosphaturia and LMWP, whereas the two female carriers presented only mild phosphaturia. A computational assessment using the VarSome [5] software, showed that the Asp631Glu variation is classified as likely pathogenic (Class 4 based on the ACMG classification), when considering the co-segregation with the disease in the family.

In order to explain the Bartter-like phenotype we also searched for relevant mutations. This search revealed only two calcium sensing receptor (*CaSR)* variations a) a homozygous common polymorphism (c.2986G > T, Ala986Ser, rs1801725, ACMG class-1) that has not been associated with Bartter-5 and b) a heterozygous rare (1/135.882) variation (c.2878C > T, Pro970Ser, rs1352202616, ACMG class-3). Three out of 4 family members with the Pro970Ser variant presented a deregulation of calcium metabolism in the form of hypercalcemia, and/or hypercalciuria (Fig. 2). However, this finding is obscured by the fact that they also carried the Asp631Glu *OCRL* mutation, which is also associated with hypercalciuria and hypercalcemia too.

## Discussion and conclusions

In 1964, Dent and Friedman were the first to describe two English males presenting with rickets, hypercalciuria and tubular proteinuria of unknown origin [6]. The term Dent disease (MIM #300009) was first introduced in 1993 to describe a form of X-linked incomplete renal Fanconi syndrome presenting with LMWP, hypercalciuria, nephrocalcinosis and nephrolithiasis and, less frequently, with aminoaciduria, phosphaturia, kaliuresis, glycosuria, uricosuria, and impaired urinary acidification [7]. In 1994, Fisher et al. cloned the *CLCN5* gene, and proposed it as a culprit for Dent disease [8]. Ten years later Hoopes et al. identified the *OCRL* gene, as responsible for the Dent disease phenotype in five males without *CLCN5* mutations and introduced the term DD2 (MIM#300555) to distinguish it from DD1 [2]. Mutations in the *OCRL* gene have been associated with both DD2 and Lowe (oculocerebrorenal) syndrome with the former being a milder form of the latter [9–12]. In 2009 one of the two original patients described by Dent and Friedman was found to carry a mutation in the *OCRL* gene [4] while the other had a *CLCN5* mutation, supporting thus the phenotypic similarities between DD1 and DD2 patients. An excellent review comparing the various presentations of DD has recently been published by Gianesello et al. [13].

Due to random X-chromosome inactivation, some female carriers may manifest hypercalciuria and, rarely, renal calculi and moderate LMW proteinuria or even CKD. In our female carries we noticed a reduced threshold for renal phosphate transport.

The males in the initial report of DD2 exhibited none of the classic extrarenal symptoms of Lowe syndrome [2], which is characterized by a) congenital cataracts, glaucoma, microphthalmia and keloids [14], b) hypotonia, delayed motor milestones, intellectual disability, and c) renal tubular involvement associated with bone disease and growth retardation [15]. Fanconi syndrome and tubular acidosis, both cardinal signs of Lowe syndrome, are rare in DD2 [9]. Hypercalciuria, nephrocalcinosis and nephrolithiasis are common in DD2 but rare in Lowe syndrome [3, 16]. The renal biopsy findings in DD2 include nephrocalcinosis, interstitial fibrosis and FSGS [17, 18].

A genotype–phenotype correlation has been suggested, because almost all severe mutations associated with Lowe syndrome are located among exons 8 and 24, while missense mutations in exons 4–15 are involved in DD2 [19].

The *OCRL* gene encodes the lipid phosphatase (OCRL1), a phosphatidylinositol 4,5-bisphosphate (PIP2) phosphatase, localized in the Golgi network, early endosomes, lysosomes and tight junctions [20]. It is involved in actin polymerization and lysosomal and endosomal membrane trafficking. In proximal tubular cells, defective recycling of the megalin receptor after endocytosis accounts for the characteristic loss of LMW proteins in Lowe syndrome and DD2 patients [21, 22].

In a mouse model of DD2 it was shown that inhibition of phosphoinositole kinase with the use of alpelisib can restore cytoskeleton and endocytosis abnormalities in tubular cells improving the renal tubular dysfunction [23].

No guidelines have been established for the treatment of Dent disease. There is a substantial risk for progressive kidney disease. The main treatment goals are to decrease hypercalciuria, prevent nephrocalcinosis and kidney stones, and delay the progression of CKD. It has been proposed that the use of thiazide may reduce hypercalciuria [24–26]. However, diuretics can deteriorate hypokalemia and volume depletion. Angiotensin-converting enzyme (ACE) inhibitors and angiotensin receptor blockers (ARB) have been used to prevent further loss of kidney function particularly in those with FSGS. In our case angiotensin blockade effectively reduced proteinuria and has probably delayed the progression of eGFR decline.

Citrate is commonly used in Lowe syndrome to treat the metabolic acidosis resulting from renal tubular acidosis. A high citrate diet has been shown to slow progression of CKD in *CLCN5* knockout mice [27] and has been used in the treatment of Dent disease; however, no human trials have proven its effectiveness and acidosis is not always present.

Growth failure can be successfully treated with human growth hormone without adversely affecting kidney function [28]. Bone disease may respond to vitamin D supplementation and phosphorus repletion in those with elevated serum alkaline phosphatase levels [29]. Vitamin D supplementation was avoided in our patient, because calcitriol levels were higher than normal and were associated with the presence of hypercalcemia, hypercalciuria and nephrocalcinosis. Contrary to vitamin D, phosphate repletion proved very successful in our case, because it not only corrected bone disease but also improved muscle strength, musculoskeletal aches, body weight and abolished hypercalciuria. There is a clinical trial (NCT02016235) going on, examining whether phosphorus repletion can reduce hypercalciuria in patients with DD1 or DD2. The results of this trial may confirm our allegations about phosphorus treatment and are eagerly awaited.

When Dent disease progress to ESRD, renal replacement therapy or kidney transplantation becomes necessary. Because DD pathogenesis is inherent to kidney defects, the disease will not recur after transplantation.

The clinical characteristics of our patient are concordant with the diagnosis of DD2. The exclusion of mutations in other relevant genes and the meticulous investigation of the clinical phenotype render the Asp631Glu amino acid substitution as the only pathogenetic possibility. It is recognized that the presence of *CLCN5* or *OCRL* mutations almost always leads to the diagnosis of Dent disease [13]. However, there is marked phenotypic heterogeneity that impedes correct diagnosis and treatment [13]. Indeed, in our proband, we noticed severe salt and potassium wasting associated with severe metabolic alkalosis, something unusual in patients with DD [13]. In a recent review of a large DD cohort (109 DD1 and 9 DD2 patients) it was noticed that a significant proportion of patients presented hypokalemia and metabolic acidosis [1]. In contrast, only six subjects (5.5%) displayed a Bartter-like syndrome with hypokalemia and metabolic alkalosis. However, in two series describing exclusively DD2 patients [2, 30] no one had metabolic acidosis, but there is no mention of metabolic alkalosis also. There are reports where both DD1 and Bartter phenotypes were seen in the same patient [31–33], but a whole genome screening was not undertaken.

It remains unclear if the Bartter-like phenotype in our proband can be solely attributed to Dent’s disease or there is a contribution by the Pro970Ser *CaSR* polymorphism [34–36]. In this respect, we have to notice that 1) all other Bartter related genes were excluded by the whole exome analysis, 2) hypocalcemia has never been recorded in carriers of Pro970Ser in our kindred, 3) phosphate repletion led to complete correction of hypercalciuria. These findings collectively preclude a pathogenetic role for the *CaSR* polymorphism or contribution of other Bartter related genes. They further support the hyper-absorptive nature of hypercalcemia and hypercalciuria which, in our case, was associated with increased calcitriol synthesis in response to primary phosphaturia and hypophosphatemia. The hyper-absorptive nature of hypercalcemia, is also supported by experimental data showing that OCRL deficiency results in an unrestricted expression of intestinal TRPV6, offering an alternative or complementary explanation for increased calcium absorption [37].

Beyond hypercalciuria, in the series of Charnas et al. the vast majority (21 out of 23) of patients with *OCRL* mutations presented dehydration with salt loss and decreased concentrating capacity [38]. Therefore, these Bartter-like features may not actually be so rare as previously thought and consist part of the DD phenotypic variability.

In conclusion we present a Family with DD2, due to a rare Asp631Glu *OCRL* mutation, with a Bartter-like phenotypic variation that could not be attributed to any Bartter associated mutations. Angiotensin blockade improved proteinuria and stabilized kidney function for several years. Finally, we showed that phosphate repletion can effectively alleviate several debilitating features of the disease and should be given early in childhood to prevent hypercalciuria and bone disease.

## Data Availability

The data that support the findings of this study are available from the corresponding author upon reasonable request. Raw data of whole exome sequencing have been submitted to clinvar@ncbi.nlm.nih.gov with a submission ID: SUB5638955.

## Abbreviations

LMWP: Low molecular weight proteinuria
CKD: Chronic kidney disease
CCN5: Chloride voltage-gated channel 5
OCRL1: Phosphatidylinositol 4,5-bisphosphate-5-phosphatase
WES: Whole exome sequencing
CASR: Calcium sensing receptor
PTH: Parathyroid hormone
TmP/GFR: Ratio of tubular maximum reabsorption of phosphate over glomerular filtration rate
cAMP: Cyclic adenosine monophosphate
NHERF1: Sodium-hydrogen exchanger regulatory factor
SLC34A1: Solute carrier family 34 member 1
SLC34A3: Solute carrier family 34 member 3
FSGS: Focal segmental glomerulosclerosis
TBMs: Tubular basement membranes
ESRD: End stage renal disease
CADD: Combined Annotation Dependent Depletion
GnomAd: Genome Aggregation Database
